# Female nursing partner choice in a population of wild house mice (*Mus musculus domesticus*)

**DOI:** 10.1186/s12983-018-0251-3

**Published:** 2018-02-20

**Authors:** Nicola Harrison, Anna K. Lindholm, Akos Dobay, Olivia Halloran, Andri Manser, Barbara König

**Affiliations:** 1 Department of Evolutionary Biology and Environmental Studies, University of Zurich, Winterthurerstrasse 190, 8057 Zurich, Switzerland; 2 Institute of Integrative Biology, University of Liverpool, Liverpool, UK

**Keywords:** Communal nursing, Female cooperation, Free-living house mice, *Mus musculus domesticus*, Pairwise relatedness, Social partner choice, Spatial genetic structure

## Abstract

**Abstract:**

**Background:**

Communal nursing in house mice is an example of cooperation where females pool litters in the same nest and indiscriminately nurse own and other offspring despite potential exploitation. The direct fitness benefits associated with communal nursing shown in laboratory studies suggest it to be a selected component of female house mice reproductive behaviour. However, past studies on communal nursing in free-living populations have debated whether it is a consequence of sharing the same nest or an active choice. Here using data from a long-term study of free-living, wild house mice we investigated individual nursing decisions and determined what factors influenced a female’s decision to nurse communally.

**Results:**

Females chose to nurse solitarily more often than expected by chance, but the likelihood of nursing solitarily decreased when females had more partners available. While finding no influence of pairwise relatedness on partner choice, we observed that females shared their social environment with genetically similar individuals, suggesting a female’s home area consisted of related females, possibly facilitating the evolution of cooperation. Within such a home area females were more likely to nest communally when the general relatedness of her available options was relatively high. Females formed communal nests with females that were familiar through previous associations and had young pups of usually less than 5 days old.

**Conclusions:**

Our findings suggest that communal nursing was not a by-product of sharing the same nesting sites, but females choose communal nursing partners from a group of genetically similar females, and ultimately the decision may then depend on the pool of options available. Social partner choice proved to be an integrated part of cooperation among females, and might allow females to reduce the conflict over number of offspring in a communal nest and milk investment towards own and other offspring. We suggest that social partner choice may be a general mechanism to stabilize costly cooperation.

**Electronic supplementary material:**

The online version of this article (10.1186/s12983-018-0251-3) contains supplementary material, which is available to authorized users.

## Background

The collective care of offspring is a key form of cooperation where individuals care for the offspring of others, and in doing so gain direct and/or indirect fitness benefits [1–4]. Within an evolutionary framework, such investment into caring for the offspring of conspecifics has been studied in a diverse range of taxa, including social insects [5], birds [6] and mammals [7]. A specific type of collective care is communal nursing where two or more reproducing females pool their litters in the same nest or burrow and indiscriminately nurse own and other offspring [8–11]. Recent evidence has demonstrated the potential for exploitation during communal nursing [12], which suggests that choice of a communal nursing partner is important. However, the mechanisms behind social partner choice within the context of cooperative care are poorly understood.

One species where communal nursing has been studied extensively in the laboratory, and less so in the wild, is the house mouse (*Mus musculus domesticus*). Laboratory studies have demonstrated that two females regularly establish egalitarian relationships in which they nurse each other’s pups non-selectively [10, 13–15]. Females pool their litters in a single nest, and for an extended period of up to 3 weeks invest in all pups present. When pups are pooled in the same nest females with pups already present are unable to distinguish own from other offspring before the onset of weaning, and cannot remove own pups again to nurse them solitarily [9, 12, 16–18]. In consequence, communal nursing has been argued to be a side effect of sharing the same social environment or nesting sites [9, 10]. In a similar vein, communal nursing has been associated with high population densities and a lack of dispersal opportunities [19].

Conversely, Weidt et al. [20] found in a free-living population that females nest solitarily despite having up to five potential communal nursing partners (another female with pups in their social environment), suggesting an element of choice in communal nursing decisions. In laboratory experiments, communal nursing has been shown to provide fitness advantages for females, such as increased lifetime reproductive success in comparison to solitary nursing. Females achieved higher success when they nursed with a related, familiar or preferred female partner [14, 18, 21]. Individual lifetime reproductive success, however, decreased below that of a solitary nursing female in groups of three or more females [11], suggesting the optimal communal nursing group size to be two females. Additionally, pups from communal nests had higher survival probabilities [9, 22], but only when offspring in the communal nest had different fathers, suggesting that the pups were better protected against male infanticide (90% of the litters raised communally were pooled with litters sired by different males; [22]). Females can also benefit from increased foraging time or time away from the nest, without affecting the amount of maternal care received by the offspring [23]. Furthermore, communal nursing may have thermoregulatory benefits, allowing pups to allocate more resources to growth, and therefore is expected to occur more frequently at higher altitudes and lower temperatures (reviewed in: [10]).

Communal nursing can therefore be considered adaptive for females, and if this is the case it raises the question of whether females use specific characteristics of a partner and/or their litters when deciding to communally nurse. Recent studies of house mice have identified milk production as a potential source of conflict between communally nursing females [12, 15]. Since females produce milk according to the total number of pups in the communal nest and not just own litter size, a female with a smaller litter overinvests in milk production in relation to her own litter size [15]. Furthermore, when the difference in birth litter size is experimentally increased, females are less inclined to nurse communally [12]. According to theoretical models and empirical evidence, exploitation costs are more often tolerated among relatives due to the indirect benefits gained [24, 25]. Such findings suggest that relatedness should be important in communal nursing decisions, and evidence in support of this has indicated that females typically nest with kin [26], and prefer partners that share allelic forms of the major histocompatibility complex (MHC) gene [27]. Green et al. [28] recently revealed that female house mice choose nesting partners who are closely related, and those who share own major urinary protein (MUP) genotype.

On the other hand, females can establish successful cooperative relationships with previously unfamiliar, unrelated partners [21, 29], and a laboratory study revealed that association during juvenile development had a stronger influence on individual lifetime reproductive success than genetic relatedness [29]. These observations suggest that other factors allow for effective communal nursing [21, 29, 30]. An earlier study found that solitary nursing occurred in about 67% of litters [20], indicating that a female should choose a partner that would avoid exploitation, allowing for mutual benefits [14, 31]. Taken together these findings imply that females may use specific cues to assess characteristics of potential communal nursing partners and/or their litters. Weidt et al. [21] demonstrated in a laboratory experiment that female house mice have increased lifetime reproductive success when nursing with a preferred female partner, suggesting that not every partner is suitable. Therefore, selection on choice of a social partner is expected to result in the evolution of specific traits that allow its bearer to gain fitness benefits through successful social interactions [32–34]. Therefore, to understand the role of partner choice in female house mice, we first need to analyse the factors involved in the decision to nurse communally.

In the present study, we investigated in detail communal nursing decisions in the natural, complex social environment of free-living house mice. Analysis of communal nursing was carried out post-hoc and did not involve manipulation of the study population. Over a period of two years, we collected information on each breeding female’s potential nursing partners and their litters. We first investigated whether communal nursing was a by-product of nest availability. We looked at whether females always nursed communally when they had the opportunity to do so, and analysed whether female decisions were independent of nest occupancy.

In a second step we analysed the factors that determine whether a female joined another female (henceforth: communal nursing decision) and focused on all situations in which a female had the choice to either join another female for communal care of litters or rear her young solitarily. Given the importance of relatedness and a population’s genetic structure in the evolution of cooperation [35–37], we analysed the genetic composition of the females’ social environment in which they exhibited choice. We further tested if communal nursing decisions were affected by the social environment, were linked to seasonal effects on reproduction, population size, or number of nest sites used by the focal female.

In a final step, we analysed social partner choice and compared traits of the chosen female with that of option females available that were not chosen as a partner for communal nursing (henceforth: social partner choice). We predicted that a female should preferentially join another female when 1) the partner is kin; 2) the chosen female is familiar, either by juvenile or peer group familiarity, or by social association through sharing the same home area; and 3) the absolute difference in own and other offspring litter size is minimized, reducing the risk of exploitation.

## Methods

### Study population

Data were collected from a free-living house mice population situated close to Zurich, Switzerland, from January 2008 until December 2009. This study period was longer than the average life expectancy of a mouse in the study population (average life expectancy: 196 d; [29]). The site was an old barn with a floor space of 72 m^2^, which was divided into four equal sections by large plastic walls (holes in these walls enabled mice to access all sections). Each section contained ten nest boxes and numerous shelters that were distributed throughout. Wooden and plastic structures provided shelter and allowed the mice to form and defend territories. The barn, although closed to larger predators, was open to dispersal and immigration of mice, and to parasites and diseases [38, 39]. Food (50/50 mixture of oats and hamster food, Landi AG, Switzerland) and water was provided ad libitum in three feeding trays and four water dispensers per section.

All individuals of the population were captured every seven weeks. Over the two-year study period on average (mean ± SE) 104.5 ± 10 adult mice, 63.6 ± 9 subadults, and 37.6 ± 10 pups (range: 0–112) were present during each population-monitoring event. Individuals weighing at least 18 g were considered adult and implanted with a subcutaneous transponder (RFID tag; Trovan-ID-100A implantable micro-transponder: 0.1 g weight, 11.5 mm length, 2.1 mm diameter; implanter Trovan-ID-100E; Euro ID Identifikationssysteme GmbH & Co, Germany). Using a one-hand technique to restrain the mice the transponder was implanted with a sterile needle in the scruff of the neck, and a tissue sample was taken from the ear for genetic analysis (ear puncher, Napox KN-293: 1.5 mm diameter). Once tagged, adults were individually identifiable allowing non-invasive monitoring of their position in the barn. Mice carrying RFID tags could either be identified with a hand-held transponder reader (during handling or when resting in nest boxes or shelters), or by an automatic antenna system that recorded the mice entering and leaving nest boxes (see below for a more detailed description). There have been no reported adverse effects of the transponders in this population or the literature. The Swiss Federal Law on Animal Protection recommends ear tissue samples for use as genetic tissue.

More detailed information about the capture procedure, the barn set-up and the population can be found in [23, 31, 40].

### Reproduction

Reproduction was closely monitored in the nest boxes, which mice could access through a single plastic entrance tube. Experimenters were able to open the nest boxes at the top, allowing any litters born to be discovered and measured. Before opening a nest box we used a hand-held transponder reader to register the identity of all tagged mice inside. All shelters were also checked for tagged adults and litters, however, females rarely gave birth to pups outside of nest boxes (for all litters observed between January 2008 and December 2009 only 7% of litters were found outside of nest boxes). Such nest checks were carried out during the day when mice were usually resting.

All nest boxes were searched for new litters every 8–12 days. These nest checks allowed us to find litters while they were still being nursed, and minimized disturbance of the nests by experimenters. As a consequence, however, litters were usually not found shortly after birth (28% of litters found during the study period were 1–3 days old, where day 1 is counted as day of birth). When a litter was found, age of the pups was determined using morphological indicators (skin pigmentation, teeth eruption, fur growth and eye opening enabled age estimation of ±1 day; [23, 31]). We further registered number of litters in the nest (1 litter = solitary, all pups of the same age; ≥ 2 litters = communal), and the size and age of each litter. When pups were estimated to be 13 days old, we took an ear tissue sample and morphological measurements. Day 13 was considered the closest age to weaning that we could safely handle and reliably locate pups, as pups open their eyes at day 14 and then attempt to escape capture (in terms of gaining independence from maternal nutrition, weaning starts at 17 days and ends at 21–23 days [13]).

### Parentage analysis

We took an ear tissue sample from every living pup when aged day 13, all handled adults and any corpses found. Following the procedure described in Auclair et al. [23], DNA was amplified at 25 microsatellite loci enabling parentage analysis assignment of mother and father for individuals. At a 95% confidence level using Cervus 3.0 [41], success at assigning a mother to pups was 87–88% over the two years studied.

### Pairwise relatedness measures and spatial genetic structure

We compared how genetically similar two individuals were to each other at 25 microsatellite loci to the average similarity between all female dyads of the year the focal female’s litter was born (either 2008 or 2009). To choose an appropriate estimator we followed methods used by Rollins et al. [42] in which pedigree *r* is compared to *r* estimated from microsatellite genotype similarity. We took from our pedigree 50 full sibling and 50 parent-offspring dyads of expected relatedness *r* = 0.5, 50 half sibling dyads (expected *r* = 0.25), and 50 dyads of unrelated individuals (expected *r* = 0; living contemporaneously and not sharing a grandparent). For all of these dyads we estimated pairwise relatedness values using five different estimates for *r* [43–47] as implemented in Coancestry 1.0 [48], and correlated them against the expected *r* values (see Additional file 1: Table S1 for summaries). From this we determined the Wang estimate [47] to have the highest correlation (R = 0.80) between expected *r* and estimated *r* (for example, full sibling expected *r*: 0.5, and estimated *r* using the Wang estimate: 0.533 ± 0.02), and therefore used this estimator to determine pairwise relatedness in the current study.

We further assessed the spatial genetic structure of females in the entire barn during the years monitored using GenAIEx 6.5 [49]. This spatial genetic autocorrelation analysis allowed comparison of genetic similarity between female mice depending on their location in the barn during nest checks. We calculated a genetic distance matrix using microsatellite genotypes. A spatial location was assigned to each female based on the nest box where she was detected, at the time of a nest check (when each nest box was scanned by a hand-held reader). To reduce autocorrelation, the first nest check in each month was used (*N* = 23 nest checks). Spatial genetic autocorrelations were computed between a focal female’s genotype and the genotypes of all other tagged females at the same location (radius of 0; starting point), and between the focal female and all tagged females recorded within increasing concentric circles of 1 m radius from the starting point. Since neighbouring nest boxes are generally located within 1 m of each other, a radius of 1 m included 1–3 nest boxes. This was repeated for all females and significance was determined by random permutations.

### Female nest box use and meetings

Every nest box had two antennas (NewBehavior AG, Zurich, Switzerland) fitted to the entrance tube allowing continuous monitoring of all tagged individuals coming and going. For a detailed description of the antenna system and remote monitoring see König et al. [40], and for an illustration of nest box stays and meetings see Additional file 1: Figure S1. Movement in and out of nest boxes by a focal female was recorded by the antenna system and analysed for a tracking period of 30 days prior to the focal female giving birth. This time period was chosen as it included the gestation of the focal female and most of the gestation and initial lactation period of potential partners (house mice gestation: 19–21 days [50]). During this 30-day period the antenna system allowed us to calculate the number of nest boxes a focal mouse visited regularly. Females use a number of neighbouring nest boxes for resting and breeding [31, 40], and we determined an individual’s home area from the nest boxes they entered. We also quantified the cumulated time a female spent in all nest boxes she entered as well as the frequency of these visits. Any nest box entered for less than 300 s within these 30 days was not considered as regularly entered and was excluded. Additionally, we determined all individuals a focal female met within this time period, the number of meetings they had, in which nest boxes they met and the total duration of these meetings (association time). This, therefore, provided a measure of recent familiarity between the focal female and each of her potential partners.

### Communal nursing and potential nursing partners

A litter is considered communal when two or more females pool their litters in the same nest box. Nest boxes were rather small (diameter of 15 cm) and we never observed two separate nests in one nest box. Litters that shared a nest box therefore were always communal. Once litters are pooled a female with pups already present in the nest is unable to discriminate between own and other offspring [9, 12, 17, 51]. Thus, for the purpose of this study we determined the decision to nurse communally as being made by the pregnant female who was about to give birth (hereafter: focal female) and not by those that had already given birth (meaning in the event a female chose to nurse solitarily, her litter could later have been joined and become communal).

Any female (hereafter: option female) that gave birth within the 16 days before a focal female gave birth was classified as a potential communal nursing partner. According to Weidt et al. [20], females only communally nurse with another female if they share the same home area, here based on shared use of nest boxes (overlapping nest box use). Thus, available options for each focal female were considered to be those litters born to females in the nest boxes regularly entered by the focal female (determined by the antenna system, as explained above). We chose sixteen days before the focal female gave birth as the limit for communal nursing options, as 16 day old pups are still nursing, but weaning starts at 17 days of age when pups begin to eat solid food [13]. Such criteria have been used in several other studies of communal nursing [12, 14, 20].

### Is communal nursing a by-product of nest availability?

The communal nursing decision of a given focal female would likely be constrained by the availability of nests within her home area. To test this we let *y* be the probability that a given female chose to form a communal nest. Let *P* be the proportion of nest boxes occupied, defined as the number of boxes in a female’s home area that already contained one or several litters during the 16 days before a focal female gave birth divided by the total number of nest boxes a female used (nest boxes considered are only those used regularly by the focal female). If all nest boxes in a female’s home area already contained litters from other females (*P = 1*), the focal female could only communally rear her litter even if her preference was to raise her young solitarily. Equally, if none of the nest boxes in her home area contained a litter (*P = 0*), starting a solitary nest would be her only option. Moreover, if the focal female randomly chose a nest box (irrespective whether occupied or not), we would expect the probability for a communal nest *y* to be directly proportional to the proportion of occupied nests, *y ~ P*. For example, if a female used 5 nest boxes, and in 2 of these boxes a litter had recently been born (*P = 0.4*), we expect a communal nest in 40% of cases, a random by-product of shared nest sites. Decisions for communal nursing will be equally random if females had individual nest box preferences and nest boxes did not vary in quality. If nests varied in quality and females preferred high quality nest boxes, on the other hand, we expect communal nursing to occur more often than randomly expected according to the options available.

To account for the constraint of nest availability on a female’s communal nursing decision, as well as to test whether communal nursing decisions deviate from random choice expectations, we modelled the probability of communal nesting *y* as a power function *P*, given as:

*y ~ P*^*a*^.

Here exponent *a* (where *a > 0*) defines the shape of the relationship. If *a = 1*, *y* will be a linear function of *P*, just as expected under random expectation. We thus used *a = 1* as our null hypothesis. If *a > 1*, the function will be convex, and females are less likely to form a communal nest then expected randomly. If *0 > a > 1*, the function will be concave, and females chose the communal nests more often than expected by chance. Also note that, as required, the function is constrained to go through point (0,0) as well as point (1,1) for all *a > 0*. In short, this analysis allowed us to test whether communal nursing was an artefact of communal living or the result of shared preferred nest boxes.

### Communal nursing decision

Our intention was to gain information about a female’s decision to nurse her litter communally with another litter. Therefore, all situations in which a female had no available option to communally nurse at the time of giving birth, in her social environment, were excluded from our analysis.

To consider an effect of population density on the decision to communally nurse in our study, we took the adult population density calculated from the population-monitoring event closest to the birth date of the focal female’s litter. To assess seasonal effects on reproduction (see [31] we included in our analyses the season the litter was born (Summer = March to August; and Winter = September to February).

**Fig. 1:**
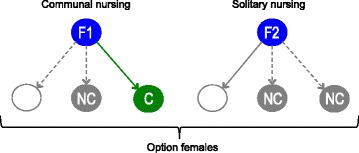
An illustration of option female categories. Blue circles illustrate the focal female, either in a scenario where she opted to join another litter and to nurse communally (F1) or where she opted to nest alone and nurse solitarily (F2). All option females are shown as either non-chosen partners (NC, grey circles) or a chosen partner (C, green circle); the white circle represents a nest where no litter was present and would have been chosen under the scenario in F2

For each option available we compiled the following data on the focal and option females, as well as their litters: association time, pairwise relatedness and age difference between the focal and the option female, age difference between the focal and option litter, and number of nest boxes the focal and option female shared. Using this dataset we determined what factors influenced a focal female’s decision to communally nurse. We had two categories of option females (Fig. 1): (1) Chosen partner (C), the option female that the focal female chose as a communal nursing partner; (2) Non-chosen partner (NC), the option female(s) that the focal did not choose to form a communal nest with; these also include the option females from the cases where the focal female chose to nurse solitarily (F2 in Fig. 1).

Litters were initially considered solitary when all pups were at the same stage of morphological development. In some cases later genetic analysis revealed “cryptic” communal nests in which two females had given birth to litters on the same day in the same nest box. In the event that a cryptic communal nest was found with no other litter, we could not be certain which female gave birth first. Therefore, we randomly chose one of the females to be the focal female, as in such a case all attributes between the two females would be equal, for example pairwise relatedness, association time and absolute age difference. In two cases a focal female joined an existing cryptic communal nest. In this scenario it was not clear which option female had been chosen, therefore, we took the average value for each characteristic.

Another predictor of choice that we considered was juvenile familiarity between a focal and an option female, which was indicated when both females were raised in the same litter (having the same mother and found in the same nest on the same day), or in the same communal nest, with a maximum age difference of 16 days. We were further interested to test the effect of litter size on the focal female’s decision to choose one option over another. However, we could not be certain that our measure of litter size for each litter was an accurate representation of birth litter size. We were only able to take the number of pups that were present when we found the litter for the first time. A more accurate measure of litter size would have required checking nest boxes daily, which would have increased disturbance and in such situations females often relocate litters elsewhere (pers. obs.). Furthermore, pregnant female house mice are known to kill pups already present in the nest before giving birth themselves [12, 14, 29, 52], which could reduce observed litter sizes.

### Social partner choice

To analyse choice of communal nursing partner we were specifically interested in the characteristics of an option female and her litter that determine whether a focal female will form a communal nest with her or not. To analyse such data we chose focal females for which we had complete information about all potential option females at that given decision from our full dataset, which reduced the data from 276 to 74 events. This reduction in data occurred because of missing parentage information of option females, or because the potential option litters were not found again at day 13 (due to pup mortality, that was likely due to infanticide [22]), therefore no genetic sample could be taken from the pups and consequently no mother could be assigned. Of these 74 we focused on the 34 occasions (*N* = 28 individual females) when a focal female chose to nest communally. We used the remaining 40 events of solitary nursing to make comparisons with the communal options.

### Statistical analysis

Statistical analyses were carried out using R version 3.1.3 [53]. Generalized linear mixed models (GLMM) were performed using the R package ‘lme4’ [54], and fulfilment of model assumptions was inspected visually from the model diagnostics [55]. To improve interpretability of parameter estimates [56] we centred and scaled the continuous explanatory variables in the appropriate models (association time, pairwise relatedness values, age difference between the females, partner age and focal female age).

We modelled the probability of choosing to nurse communally as power function of the proportion of occupied nests (*y ~ P*^*a*^, see above) using a non-linear least squares model (NLS). This approach allowed us to find the best estimates for shape parameter *a* given our observed data and, equivalently, to test whether communal nursing was an artefact of communal living. We analysed the probability of a focal female choosing to communally nurse vs. solitarily nurse using a binomial GLMM (option taken = 1, option not taken = 0). We specified a full model which included the following fixed effects: number of options available, number of nest boxes entered, age of the focal female, experience of focal female (whether the focal female had a litter before), adult population density, and season. Female identity was included as a random effect.

We further assessed the probability of an option female being chosen using a binomial GLMM (chosen female = 1, non-chosen female = 0). The fixed effects in our full model included: age of the option female’s pups on the day the focal female’s pups were born, litter size difference (absolute), number of nest boxes shared, age difference between the focal and option female, age of option female, pairwise relatedness, association time with the option female, and whether the option litter was solitary. Event ID (a unique number given to each decision), focal female identity and option female identity were included as random effects. We were unable to specifically test juvenile familiarity due to an incomplete data set; we therefore looked at cases where the information was available to assess its occurrence, and used age difference between the females as a proxy.

We carried out initial model selection on both GLMMs to determine whether the interaction terms were important by using the model selection function in the MuMIn package [57]. Our models were compared to all possible combinations of that model containing the same or fewer interaction terms, and to a model containing no interactions, and all fixed effects were kept in the model. Models were ranked by corrected Akaike information criteria (AICc), whereby the model with the lowest AICc value was chosen to be the most adequate model. In the event that two or more models fell within 2 delta AICc of each other, we then chose the model with the lowest degrees of freedom. In both cases the most adequate model was the model containing no interaction terms, and therefore all interactions were considered non-significant for *P* > 0.05. To determine the significance of each fixed effect, we compared a model with the fixed effect of interest removed to the model containing all fixed effects, using likelihood ratio tests [58, 59]. All random effects were kept in the model and variance components were estimated using maximum likelihood methods (“ML”).

To test the difference between the pairwise relatedness of the focal female to the option female(s) in the communal nursing scenario and in the solitary nursing scenario, we used a linear mixed model (LMM), with event ID, focal female identity and option female identity included as random effects. To determine whether the communal options and the solitary options differed with regard to age of pups, we used a Wilcoxon-Mann-Whitney rank sum test, as in this case the residuals were not normally distributed (tested using a quantile-quantile plot; [58]).

## Results

**Fig. 2:**
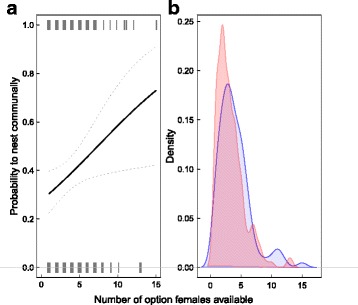
**a** Probability of a focal female choosing to nurse communally (score = 1) vs. solitarily (score = 0), here shown against the number of potential communal nursing partners available at a given decision. Tick marks demonstrate the variability in the number of options available. **b** Density plots of the number of available options for the females that chose communal (blue) and the females that chose solitary (red)

During the two-year study period we collated information on 314 litters producing 1432 pups. All litters raised communally confirmed previous observations [20] that their mothers had shared nest boxes before giving birth, thus validating our definition of option females (see Methods). In 276 litters (*N* = 127 individual females) the focal female had at least one option to nest communally. In the remaining 38 cases (*N* = 34 individual females) no other female gave birth in the previous 16 days, meaning they had no option but to nest solitarily (these were excluded as focal females). Within a female’s home area, the number of females considered as an option (altogether *N* = 128 individual females) ranged from 1 to 15 (mean ± SE: 3.55 ± 0.14 available options; Fig. 2). Females chose to nest communally in 106 cases (38.4%; *N* = 77 individual females) and solitarily in 170 cases (61.6%; *N* = 98 individual females); 48 females used both nursing strategies during the study period. Females on average used 5.20 ± 0.10 (mean ± SE) nest boxes and interacted with a total of 8.69 ± 0.24 (mean ± SE) tagged females (includes all adult female interaction partners of the focal female during the 30-days prior to birth, reproducing and non-reproducing).

### Is communal nursing a by-product of nest availability?

**Fig. 3:**
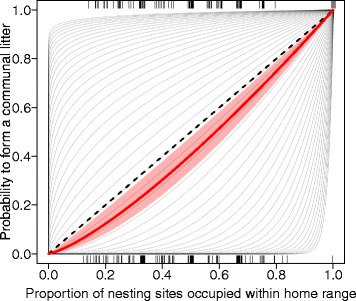
Nest availability analysis: dashed line represents *a* = 1 suggesting choice of partner was equal to the proportion of occupied nesting sites (nest boxes containing one or several litters), if *a* < 1 females choose communal (1) more often than random expectation and, if *a* > 1 females choose solitary (0) more often than random. Potential values for *a* are represented by the light grey lines. For the raw data we find a value of *a* = 1.3, here represented by the red line (± 95% CI polygon). Tick marks represent the variability in the proportion of occupied sites

Results from the nest availability analysis of communal nursing decisions indicated that our observed data differed significantly from random expectation based on nest box occupancy (t = 2.57, *P* = 0.014, null expectation: *a* = 1, observed data: *a* = 1.30, Fig. 3), which suggested females chose to nurse communally less frequently than under random expectation.

### Communal nursing decision

**Fig. 4:**
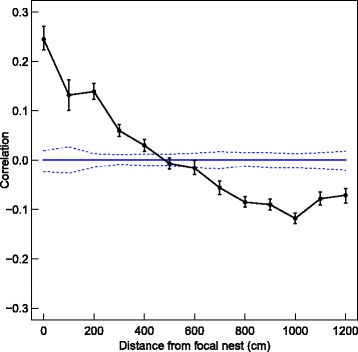
A plot illustrating the genetic spatial structure analysis for all the females in the study population, during 2008 and 2009. Estimates and 95% CI are shown per distance class (in black). Blue line represents the average (zero) with a 95% CI, illustrating the null hypothesis of no spatial structure. All confidence intervals were obtained by bootstrapping

We initially investigated the genetic spatial structure of the females for the two years that the study population was monitored, and found that the relatedness between adult females was positively correlated when females were found in the same nest box (0 cm) and up to 400 cm from that nest box (significant positive genetic spatial structure was observed at: 0, 100, 200, 300 and 400 cm, *P* < 0.001, Fig. 4). As distance from the central point increased, these correlations declined to zero and below, implying that female mice were found close to genetically similar individuals.

Adult population density (taken from the closest population-monitoring event) did not significantly affect the decision to nurse communally (Table 1). More litters were born in summer (March to August; *N* = 228; communal = 90, solitary = 138) than in winter (September to February; *N* = 48; communal = 15, solitary = 33), but there was no significant influence of season.

**Table 1:** GLMM results analysing the decision to nurse communally and the choice of communal nursing partner

Response variable	Explanatory variables	Estimate	95% CI Lower, Upper	Likelihood ratio test (χ^2^)	*P*
Decision to nurse communally	**Number of options available**	**0.14**	**0.01, 0.26**	**4.89**	**0.027**
	Number of nest boxes entered	−0.07	−0.24, 0.10	0.66	0.418
	Age of the focal female	0.10	−0.21, 0.41	0.42	0.518
	Experience of focal female	−0.54	−1.20, 0.12	2.59	0.108
	Adult population density	0.28	−0.43, 0.99	0.58	0.447
	Season	−0.26	−0.97, 0.46	0.51	0.477
Choice of partner	**Age of option pups**	**−0.44**	−**0.66, − 0.22**	**26.92**	**< 0.001**
	Litter size difference	−0.32	−1.05,0.40	0.75	0.387
	Number of nest boxes shared	−0.19	−0.90, 0.53	0.27	0.606
	Age difference between the females	−0.57	−1.23, 0.09	2.90	0.088
	Age of option female	−0.20	−0.90, 0.49	0.33	0.565
	Pairwise relatedness	0.12	−0.56, 0.80	0.11	0.738
	**Association time**	**0.95**	**0.01, 1.89**	**4.39**	**0.036**
	Was option litter solitary?	−0.24	−1.72, 1.23	0.10	0.749

*Explanations*: Decision to nurse communally = whether the focal female chose to form a communal nest (1) or whether she decided to nurse solitarily (0); Choice of partner = whether the option female was chosen (1) or not (0); Age of the focal female, Age of option female (potential partner) and Age difference (absolute) between the females = calculated as the age the mouse would have been at the time the focal female gave birth to her litter; Experience of focal female = whether a focal female had a litter previously or not (whereby 1 = yes and 0 = no, first litter); Age of option pups = age of the option female’s litter at the time the focal female gave birth. Significant factors (*P* < 0.05) are highlighted in bold

Focal females were significantly more likely to nurse their litters communally with an increasing number of available options in their home area (Fig. 2a, Table 1). Unsurprisingly, the number of options available to a female increased with number of nest boxes used by the focal female, but this had no significant effect on her decision (Table 1). Females sometimes chose to nurse solitarily even when there were up to 13 potential partners available. Additionally, there was no significant effect of reproductive experience of the focal female (whether she had a litter before; Table 1) on her decision to nurse communally.

### Social partner choice

The decision to choose one partner over another was not significantly influenced by pairwise relatedness (Tables 1 and 2). However, there was a significant difference between relatedness of all option females in the communal scenario (overall mean of all option females when a focal chose communal nursing: *r* = 0.235 ± 0.02) and those of all the option females in the solitary scenario (mean: *r* = 0.192 ± 0.02; ${\chi}_1^2$ = 4.57, *P* = 0.033). A female thus chose to nurse communally when her available options were generally more closely related to her.

**Table 2:** Attributes of the option females under the different scenarios, whether the partner was chosen as a communal nursing partner or not

Attributes of the option females	Focal chose CN		Focal chose SN
	Chosen	Non-chosen	Non-chosen
Age of pups (d)	2.7 ± 0.5	8.0 ± 0.7	6.6 ± 0.6
Litter size difference	3.7 ± 0.7	2.9 ± 0.5	2.2 ± 0.1
Number of boxes shared	2.7 ± 0.2	2.7 ± 0.2	2.3 ± 0.2
Age difference between females (d)	141.8 ± 24.4	190.7 ± 28.6	136.5 ± 16.4
Age of option female	297.2 ± 23.8	301.0 ± 30.8	336.3 ± 16.6
Pairwise relatedness	0.30 ± 0.04	0.26 ± 0.03	0.19 ± 0.04
Association time (min)	4303.1 ± 720.3	2098.3 ± 402.4	2458.0 ± 368.9

Attributes are given as mean ± SE. *CN* Communal nursing, *SN* Solitary nursing; Litter size difference, the absolute difference between focal female litter size and the option female’s litter size; Age difference between females is given as absolute days, and is calculated from the date the focal female gave birth

The likelihood of an option female being chosen significantly increased when her pups were young (Table 2). 82% of the focal females joined a litter that was maximally 5 days old, and 72% of the females chose a litter that was younger than the average of her other available option litters (all females that chose to nurse communally from the full data set, *N* = 105). To test if age of pups influenced a focal female’s decision to nurse solitarily, we compared the age of pups in the option litters available to a focal female that chose solitary nursing against the age of pups in the option litters available to the communally nursing females (Table 2). We found a non-significant tendency for the available litters in the communal nursing scenario to be younger (Wilcoxon test; *W* = 2981, *P* = 0.074), suggesting that the availability of a young litter may have played a role in a focal female’s decision.

Focal females spent significantly more time with the chosen partner in the month before birth than with her other option females (Tables 1 and 2), suggesting the chosen partner was more familiar. We then explored the potential for juvenile familiarity to play a role in partner choice. Only 18.8% of option females (27 of 148 option females) were of a similar age to the focal female (differing by a maximum of 16 days in age). Twelve of these 27 were females born in the same litter (siblings) or in the same communal nest as the focal female. Of the focal females that chose to nurse communally only 6 had the option to choose a litter sibling, of which in only 2 cases they chose her as a partner (one female chose a maternal sibling and the other a full sibling). Therefore, only a few focal females (12.6%) in this data set had the option to raise a litter communally with a partner that was from the same litter, and therefore familiar by juvenile association. Furthermore, there was no significant effect of age difference between the focal and the option female (Additional file 1: Figure. S2), or the option female’s age on the focal female’s decision (Tables 1 and 2). The decision to choose one partner over another was not significantly influenced by absolute difference in litter size.

## Discussion

Free-living female house mice were ‘choosy’ in their decision to nurse communally, as they did not always communally nurse when they had the opportunity. Adult females shared nest boxes and regularly met with on average only 8–9 females (including non-breeding tagged females) in their overlapping home area, and thus seemed to establish fairly closed social groups. Within such groups, pregnant females had on average 3–4 options to join another litter for communal nursing, but more often chose to nurse their litters solitarily, a decision that was more likely than random expectation. They did so even when there were up to thirteen potential communal nursing partners available in their home area. Adult population density did not affect a female’s probability to choose communal nursing. Hence, these observations do not support the hypothesis that communal nursing was a by-product of sharing the same nesting sites. On the contrary, they reinforce results observed by Weidt et al. [20], who studied the same population 5 years earlier when the population size was much lower (maximal density: 0.94 adults / m^2^), and comprised only 36% of the maximum population density analysed in this study (minimum: 0.72 adults / m^2^; maximum: 2.61 adults / m^2^).

The probability that a focal female chose communal nursing increased with the number of potential partners, suggesting that the probability of a preferred communal nursing partner being available increased when more options were accessible. However, this was not a side effect of more nesting sites being occupied as our nest availability analysis determined that females chose communal nursing less often than random expectation. This supports our initial expectation that social partner choice is an important aspect of female cooperation. Most interestingly, choice was exhibited in a social environment that was composed of genetically similar individuals (a female’s home area on average consisted of relatives), and within such social groups females chose partners that were familiar through previous association and had recently given birth.

### Social partner choice – Relatedness versus familiarity

According to kin selection theory relatedness is required for the evolution of costly cooperation [35, 36, 60–62], and is invoked to explain cooperative behaviour not only in vertebrates (birds: [6]; mammals: [3]), but also in social insects [63, 64], and bacteria [65]. We found no effect of pairwise relatedness on the decision to nurse with one partner over another, as we had expected. Females may have been less inclined to fine-tune their discrimination [35, 36, 66, 67] given that the females in their home area were on average genetically similar to them. This would mean that investment into another female’s offspring during communal nursing could be compensated by the indirect fitness benefits gained. Exploitation costs are also more often tolerated when cooperating with a relative [24, 25], which is supported by our observation that focal females chose to communally nurse more often when the general pairwise relatedness of their options was higher. Alternatively, it is possible that mice assess relatedness at specific loci instead of genome wide, particularly at the MHC and MUP genes [27, 28, 68]. Therefore, in our study population it is possible that overall genetic relatedness is a less important cue than MHC or MUP similarity; future studies could investigate such differences and their influence on communal nursing decisions.

Juvenile familiarity, arising from females having been raised together in the same nest, was shown to have major importance in laboratory studies with wild-bred house mice [29]. Females had increased offspring survival with a familiar unrelated partner they grew up with over an unfamiliar sibling. In our study, we observed that females very rarely had the opportunity to communally nurse with a litter sibling. Such lack of opportunities can be explained by the low average life expectancies in house mice [69], high pup mortality [22], reproductive skew [31], or the possibility of dispersal within and from the study population. Even if a female shared a home area with a litter sibling, we predict that the chance of both females having litters within such a short period of time is likely to be low. Here, we can rule out juvenile familiarity as a decisive factor, since only 12.6% of females had a familiar partner available in her choice set and only 6% of focal females chose a sister from her birth litter. We further found that females did not discriminate by age of the option female. If juvenile familiarity were important we would have expected a female to prefer those that were similar in age to her.

Females chose a partner with whom they had associated most often during the month before giving birth, when controlling for age differences between litters, which is a measure of birth synchrony. We suggest that association time in nest boxes reflects individual preferences among females prior to reproduction, resulting in familiarity with the partner. In support of our suggestion, Weidt et al. [20], although using a different measure for association, demonstrated that individual associations established during the non-reproductive period predicted choice of a nursing partner. Females chose a communal nursing partner with whom they had a higher dyadic association when both had been non-breeding, suggesting that females establish preferences prior to reproduction.

Choice of a communal nursing partner may also have occurred under a hierarchy of cues [70]. In a mate choice study, female mice demonstrated complexity in their decision-making, whereby their choice was affected by the variance in different characteristics of males [68]. MHC dissimilarity predicted mate choice only when the variability in male scent marking rates (high scent marking indicates a higher dominance rank) was low [68]. Females in the context of communal nursing decisions may also adopt such a strategy by using alternative cues to choose a partner from a group of females when the variance in pairwise relatedness is low. An additional hypothesis could be that relatedness facilitates establishing associations among females that allow for communal nursing, and therefore the absence of a kinship effect could be explained by the existing social associations between females. This may also suggest that when females live among unrelated females they do not have these associations, leading to a reduced propensity to cooperate via communal nursing.

### Social partner choice – Influence of litter size

Contrary to our prediction, litter size did not play a significant role in explaining choice of a communal nursing partner. An experimental study showed that females avoid communal nursing when litter sizes at birth are unequal [12], however, in the free-living environment litter size does not appear to play a role in communal nursing decisions. One explanation may be that our measure of litter size at the time of discovery of the pups differs from litter size at birth. Pregnant females are known to manipulate a partner’s litter by killing one or several of her pups before giving birth themselves, resulting in communally nursed litters being smaller (by 1–2 pups) than those nursed solitarily [12, 14, 23, 29, 52]. Such infanticide would result in a reduction of litter size prior to litters being found, which could potentially have hidden an effect.

### Why do females prefer young pups for communal care?

Focal females preferred to join another female when her partner’s pups were young, with 82% of these being less than or equal to five days old. This finding compliments those of Weidt et al. [9], who found females were more likely to choose nests for communal care with pups younger than the average of the other available litters. Females may have chosen a younger litter to prolong the period of time that the partner female was unable to discriminate between offspring, and increase the chance both litters would be nursed equally. Some studies have shown that females are more likely to discriminate between offspring in a communal nest when the age disparity between them is larger [9, 10, 71, 72]. In house mice, however, it is believed that females cannot discriminate between own and other offspring when mixed in one nest before the onset of weaning [9, 12, 17, 51], or at least have a limited ability to do so [73]. Effective nest defence is likely highest in the days following birth, and postpartum aggression in females during this time is highest during the first 3 days, in particular towards unfamiliar intruders [74]. Therefore, joining a familiar female with young pups could ensure high nest defence, reducing the chance of infanticide by intruders. Furthermore, although inter-litter competition has only been a topic of speculation in house mice [10, 75], competition between pups should be less costly when the age between the two litters is smaller [10]. Females may have been attempting to avoid inter-litter competition, and therefore promoted own offspring survival by choosing young litters. To better understand the reasons behind choice of a litter with young pups, and disentangle other factors such as familiarity or relatedness among social partners, a different data set or use of empirical studies is required where factors can be controlled and experimentally modified, or where fitness consequences can be assessed.

## Conclusions

Taken together, our findings show that communal nursing is not a by-product of sharing the same nesting sites, and that female house mice have the capacity to choose a communal nursing partner from a social group of genetically similar females, and in doing so preferred those that are familiar and have young pups. We suggest that such social partner choice evolved because of the risk of exploitation during communal nursing and allows females to cooperate in the presence of conflict.

## Additional file

Additional file 1: Supplementary materials. Included are a supplementary table (**Table S1**) and two supplementary figures (**Figures S1-S2**). (DOCX 2196 kb)
