# Supplementary material for: Female nursing partner choice in a population of wild house mice (Mus musculus domesticus)
Source: Front Zool. 2018 Feb 20;15:4. doi: 10.1186/s12983-018-0251-3 (PMC5819181; doi:10.1186/s12983-018-0251-3)
Supplement: Supplementary file 1 — Supplementary materials. Included are a supplementary table (Table S1) and two supplementary figures (Figures S1-S2). (DOCX 2196 kb) [file 12983_2018_251_MOESM1_ESM.docx]

**Supplementary Material**

**Table S1**. Expected and average *r* values for five estimators of relatedness among pairs of females.

| Estimator | Unrelated | Full Sibling | Half Sibling | Parent-offspring | Correlation |
| --- | --- | --- | --- | --- | --- |
| Expected | 0 | 0.5 | 0.25 | 0.5 | -- |
| West | -0.041±0.03 | 0.533±0.02 | 0.283±0.03 | 0.495±0.02 | 0.800 |
| LLEst | -0.040±0.03 | 0.539±0.02 | 0.284±0.03 | 0.495±0.02 | 0.791 |
| LREst | -0.016±0.02 | 0.503±0.03 | 0.274±0.03 | 0.480±0.02 | 0.789 |
| Rest | -0.009±0.02 | 0.533±0.04 | 0.232±0.04 | 0.492±0.05 | 0.662 |
| QGEst | -0.030±0.02 | 0.545±0.02 | 0.294±0.02 | 0.493±0.02 | 0.799 |

The estimated *r* values are given as mean ± SE. Estimators: West [47]; LLEst [44]; LREst [46]; Rest [45]; QGEst [43]. Correlation here stated as the correlation between the expected *r* value and the estimated *r* for each estimator tested.

**Figure S1**. An illustration of nest box stays and meetings. **(a)** Birds eye view of a nest box in the barn (here box 2), the white square indicates the nest box lid (ceramic tile), the two black boxes indicated by the letters A and B are the outer (A) and inner (B) antenna, which are attached to a clear acrylic entrance tube (this photograph illustrates our new antenna system (AniLoc system, FBI Science GmbH, Germany), however, the position of the antennas and method of reading transponders was the same as the previous system, NewBehavior AG, Zurich, Switzerland). **(b)** Two antennas are required in order to distinguish between a mouse entering and leaving a box, which allowed us to determine a nest box stay. When a mouse entered a box it was read first by antenna A followed shortly after by antenna B, and vice versa when it left the box (each nest box had two unique identifiers for the antennas). The time (s) between the ‘in’ and ‘out’ readings was classified as a nest box stay; **(c)** Illustrates how nest box meetings between different individuals were monitored, classified by the time (s) in which 2 mice (mouse 1 (M1) and mouse 2 (M2)) overlap inside the nest box, we can also calculate the number of times they meet (counts).

**Figure S2**. Plot to illustrate the distribution of absolute age difference (in days) between a focal female and all her available options (irrespective of whether an option was chosen or not). The solid line indicates the mean age difference for all options (152.9 d), and the dashed line represents the mean age difference for the chosen option females only (141.8 d). This figure demonstrates that although right-skewed towards smaller age differences the majority of options had an age difference of more than 20 d, and age difference varied.
